# Supplementary material for: Brain cross‐protection against SARS‐CoV‐2 variants by a lentiviral vaccine in new transgenic mice
Source: EMBO Mol Med. 2021 Oct 25;13(12):e14459. doi: 10.15252/emmm.202114459 (PMC8646827; doi:10.15252/emmm.202114459)
Supplement: Supplementary file 1 — Appendix [file EMMM-13-e14459-s002.pdf]

Appendix

Appendix Figure S1. Comparative description of the hACE2 constructs used to generate B6.K18-hACE2^{IP-THV} and B6.K18-ACE2$^{Prlmn/JAX}$ transgenic mice and their features after inoculation of SARS-CoV-2.

Appendix Figure S2. Wild-type and prefusion forms of S_{CoV-2} protein and comparative antibody responses of mice immunized with LV encoding each of these forms.

Appendix Figure S3. Additional immune features of B6.K18-hACE2^{IP-THV} mice primed (i.m.) and boosted (i.n.) with LV::S.

Appendix Figure S4. Map of lentiviral plasmid encoding for S_{CoV-2}.

Appendix Table S1. S_{CoV-2}-derived murine and human T-cell epitopes.

Appendix Table S2. Sequences of prefusion S_{CoV-2}, as encoded by LV vaccinal vectors.

Appendix Table S3. Sequences of primers used to genotype B6.K18-hACE2^{IP-THV} transgenic mice.

Appendix Table S4. Sequences of primers used to quantitate SARS-CoV-2 RNA content by qRT-PCR.

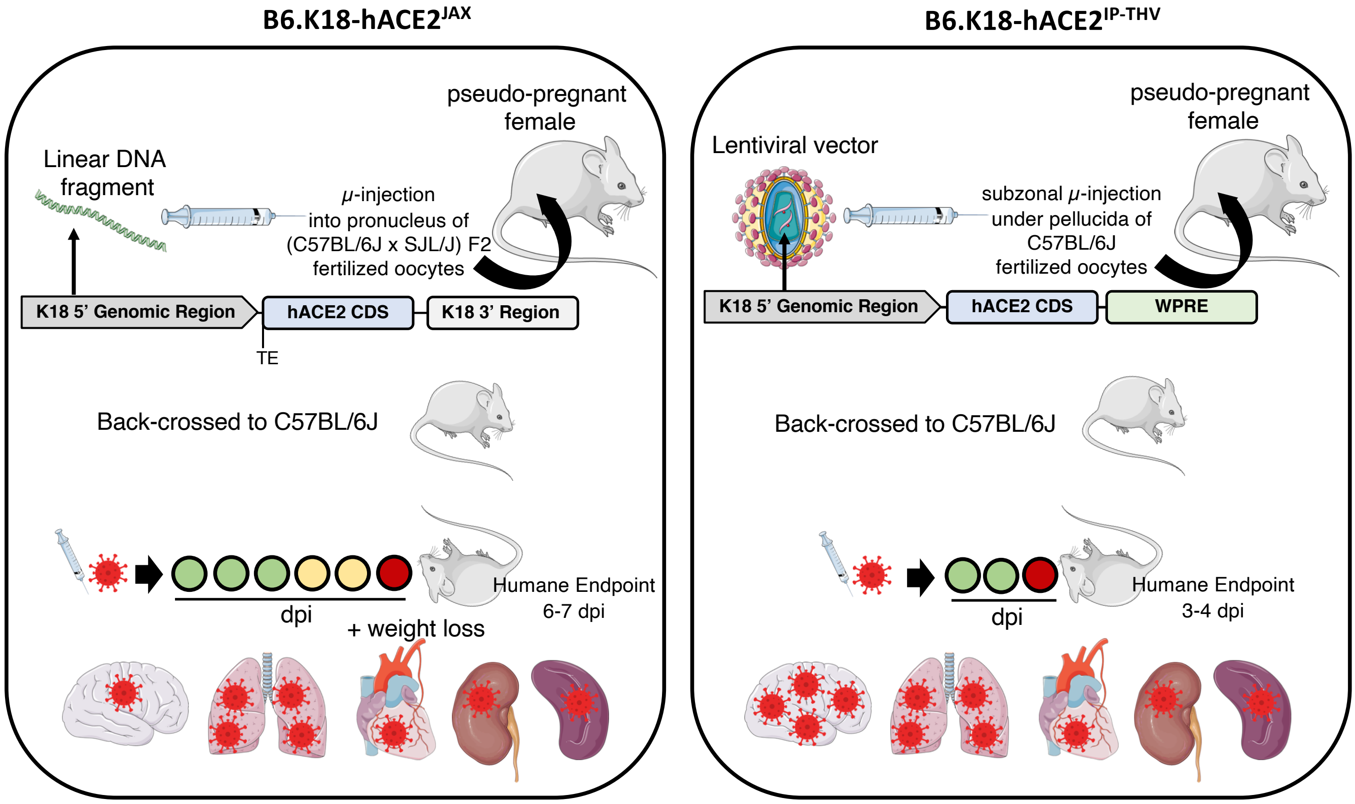

Appendix Figure S1. Comparative description of the hACE2 constructs used to generate B6.K18-hACE2$^{IP\text{-}THV}$ and B6.K18-ACE2$^{Prlmn/JAX}$ transgenic mice and their features after inoculation of SARS-CoV-2. The characteristics of B6.K18-ACE2$^{Prlmn/JAX}$ mice are based on the previous description (McCray *et al*, 2007; Winkler *et al*, 2020). The characteristics of B6.K18-hACE2$^{IP\text{-}THV}$ mice are based on the results in the present work. K18 = human cytokeratin 18 promoter, CDS = Coding DNA Sequence, TE = Translational Enhancer from alfalfa mosaic virus, WPRE = Woodchuck Posttranscriptional Regulatory Element translational enhancer.

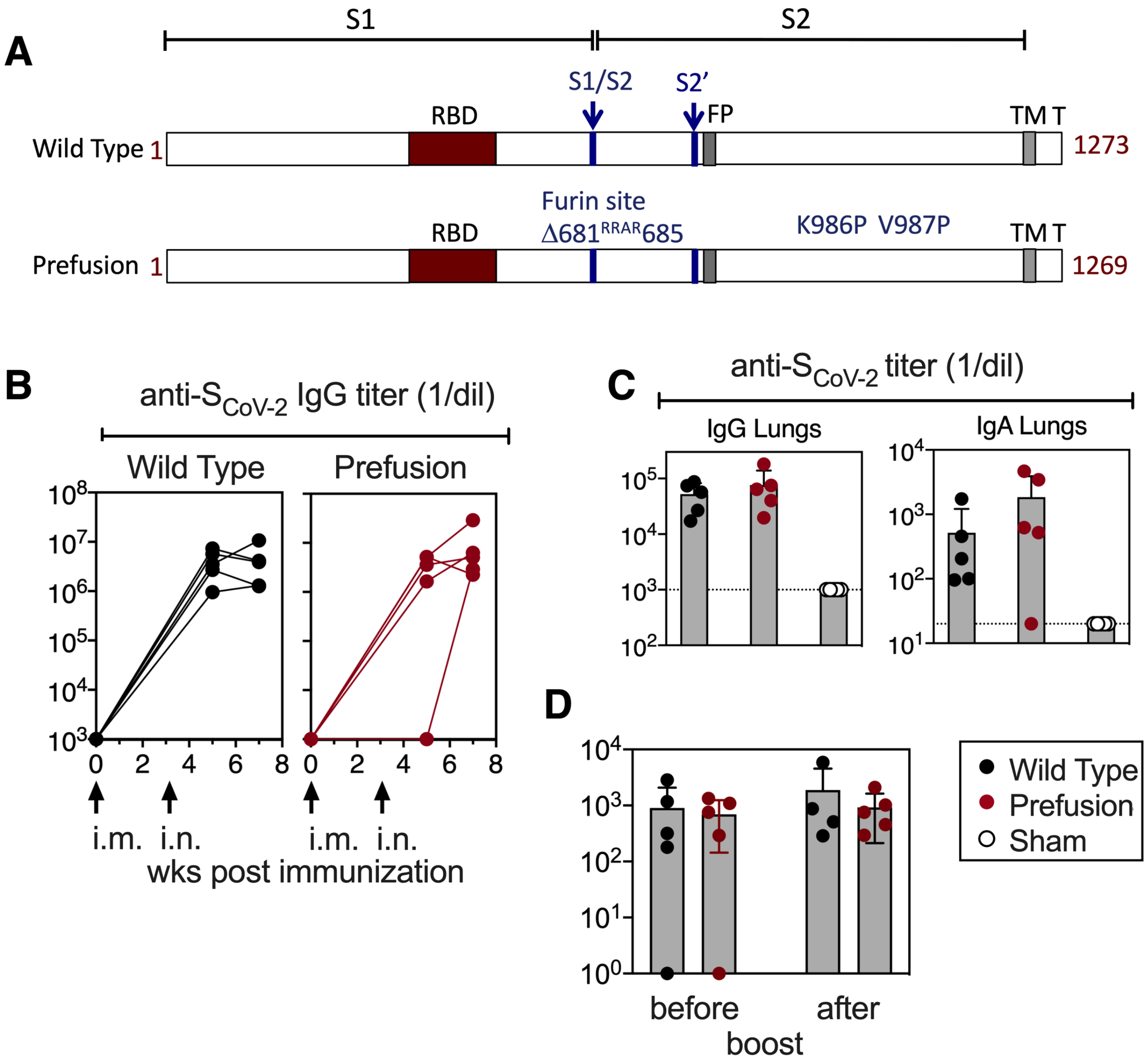

Appendix Figure S2. Wild-type and prefusion forms of S_{CoV-2} and comparative adaptive immune responses of mice immunized with LV encoding each of these forms. (A) Schematic representation of wild type or prefusion forms of S_{CoV-2} encoded by LV. RBD, S1/S2 and S2' cleavage sites, Fusion Peptide (FP), TransMembrane domain (TM) and short internal tail (T), $675^{QTQTNSPRRAR}685$ sequence encompassing RRAR furin cleavage site, and $K^{986}P$ and $V^{987}P$ consecutive substitutions are indicated. **(B-C)** C57BL/6 mice were primed i.m. at wk0 with 1×10^7 TU and boosted i.n. at wk5 with 3×10^7 TU of either of LV or a control LV (sham). The LVs used in this experiment were integrative. **(B)** Sera were collected at 3, 5 and 7 wks post immunization and anti-S_{CoV-2} (TriS) IgG responses were evaluated by ELISA. Results are expressed as mean endpoint dilution titers. **(C)** Lung homogenates were studied at 7 wks post immunization for anti-S_{CoV-2} (TriS) IgG or IgA responses. **(D)** Comparative serum neutralization capacity of anti-S_{CoV-2} sera induced by LV immunization, determined as 50% Effective Concentration (EC50) neutralizing titers. Shown are Mean ± SD.

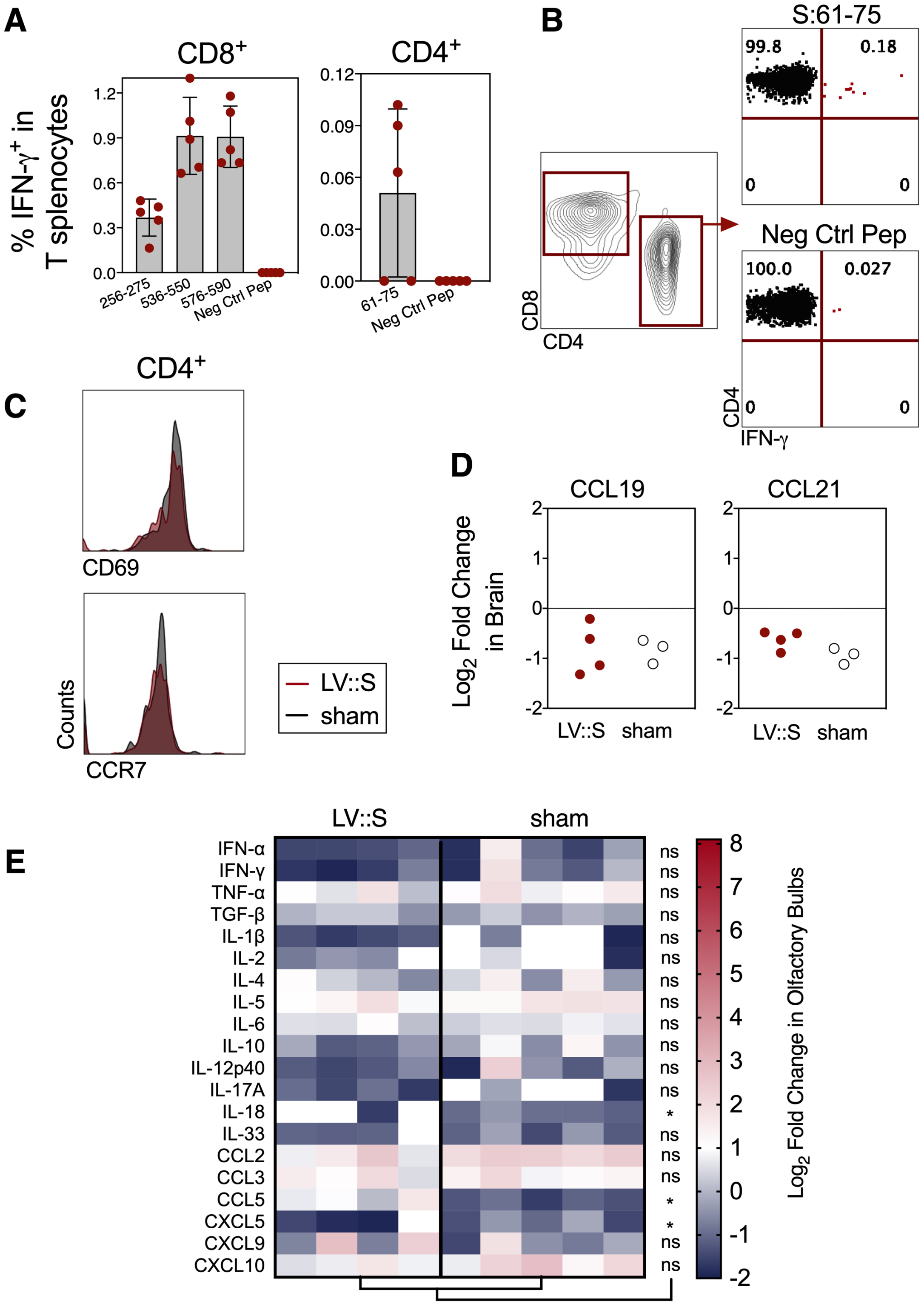

Appendix Figure S3. Additional immune features of B6.K18-hACE2^{IP-THV} mice primed (i.m.) and boosted (i.n.) with LV::S. (A) IFN-γ T splenocyte responses quantified by ELISPOT in the LV::S-vaccinated B6.K18-hACE2^{IP-THV} mice, using various 15-mer peptides encompassing S_{CoV-2} MHC-I- (left) or MHC-II- (right) restricted T-cell epitopes. **(B)** Representative results of intracellular staining of IFN- γ to measure lung CD4^{+} T cells, after in vitro stimulation with S_{CoV-2}::61-75 peptide containing an MHC-II- restricted T-cell epitope. **(C)** Cytometric analysis of CD69 and CCR7 expression on CD3^{+} CD4^{+} T cells gated on cells extracted from pooled olfactory bulbs of B6.K18-hACE2^{IP-THV} mice vaccinated with LV::S or sham at 3 dpi after SARS-CoV-2 challenge. **(D)** Expression of CCL19 or CCL21 chemo-attractants in the brain, as evaluated by qRT-PCR at 3 dpi. **(E)** Heatmap representing $\log_2$ fold change in cytokine and chemokine mRNA expression in the olfactory bulbs, from LV::S-vaccinated ($n = 4$) and sham ($n = 5$) individuals, at 3 dpi. Data were normalized versus untreated, non-infected and age-matched controls. Statistical significance was evaluated by Mann-Whitney test (*= $p < 0.05$, ns = not significant). The scale of the heatmap is identical to that of Figure 3C and EV4 to facilitate comparison of the degrees of inflammation in various organs studied.

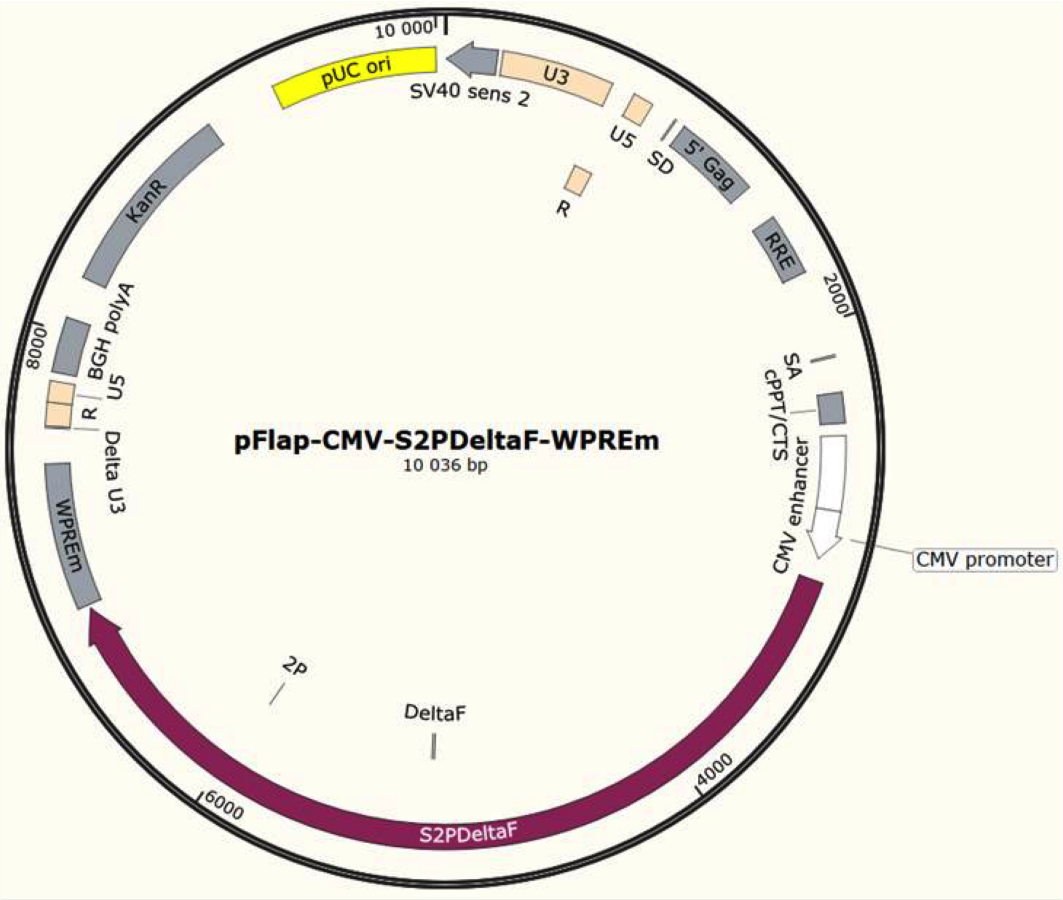

Appendix Figure S4. Map of lentiviral plasmid encoding for S_{CoV-2}. The Full-length ancestral S_{CoV-2} sequence is depleted for $675^{QTQTNSPRRAR}685$ sequence, encompassing the RRAR furin cleavage site, and harbors $K^{986}P$ and $V^{987}P$ consecutive substitutions, as indicated on the map. WPREm = mutated Woodchuck Posttranscriptional Regulatory Element translational enhancer.

Appendix Table S1. $S_{CoV\text{-}2}$-derived murine and human T-cell epitopes.

Murine	Sequence aa	a.a substitution / deletion
H-2D^b	LDS**KVGGNYNYL**YRL	**L** deleted in Delta
H-2D^b	NK**CVNFNFNGL**TGTG	
H-2D^b	V**RDPQTLEIL**DITPC	
H-2D^b	CASY**QTQTNSPRRA**R	**P** —> H in Alpha, **P** —> R in Delta
H-2D^b	VQID**RLITGRLQSL**Q	
**Human**	**Identified (Immundex data base)**	**observation**
A*0101	LTDEMIAQY	
A*0201	FLHVTYVPA	
A*0201	KIYSKHTPI	
A*0201	KLPDDFTGCV	
A*0201	LLFNKVTLA	
A*0201	RLDKVEAEV	
A*0201	RLITGRLQSL	
A*0201	RLQSLQTYV	
A*0201	TLDSKTQSL	
A*0201	VLNDIL**S**RL	**S** —>A in Alpha
A*0201	YLQPRTFLL	
A*0201	RLNEVAKNL	
A*0201	VVFLHVTYV	
A*0201	NLNESLIDL	
A*0201	FIAGLIAIV	
A*0301	KCYGVSPTK	
A*0301	GVYFASTEK	
A*1101	RLFRKSNLK	
A*1101	GTHWFVTQR	
A*1101	GVYFASTEK	
A*2402	KWPWYIWLGF	
A*2402	QYIKWPWYI	
A*2402	NYNYLYRLF	**L** —> R in Delta
A*2402	RF**D**NPVLPF	**D** —> A in Beta
B*0702	S**P**RRARSVA	**P** —> H in Alpha, **P** —> R in Delta
B*0702	APHGVVFL	
B*3501	QPTESIVRF	
B*3501	LPFNDGVYF	
B*3501	IPFAMQMAY	
B*4403	YEQYIKWPW	
DR	ITRFQTL**LAL**HRSYL	**LAL** deletion in Beta
DR	FNGLTVLPPLLTDEM	
DRB1*0101<br>DRB1*0401<br>DRB1*0701<br>DRB1*1501	QLIRAAEIRASANLA**A**TK	**A** —> I in Gamma

Murine CD8$^+$-T cell epitopes have been previously identified (Ku *et al*, 2021). Human T-cell epitopes are from the updated Immudex data base (https://www.immudex.com/media/1535/tf119203-published-list-of-covid-19-t-cell-epitopes.pdf). Red a.a. are those substituted or deleted by mutations occurred in the $S_{CoV\text{-}2}$ of Alpha, Beta, Gamma or Delta SARS-CoV-2 variants of concern.

Appendix Table S2. Sequences of prefusion S_{CoV-2}, as encoded by LV vaccinal vectors

**Prefusion a.a. sequence**
MFVFLVLLPLVSSQCVNLTTRTQLPPAYTNSFTRGVYYPDKVFRSSVLHSTQDLFLPFFS<br>NVTWFHAIHVSGTNGTKRFDNPVLPFNDGVYFASTEKSNIIRGWIFGTTLDSKTQSLLIV<br>NNATNVVIKVCEFQFCNDPFLGVYYHKNNKSWMESEFRVYSSANNCTFEYVSQPFLMD<br>LEGKQGNFKNLREFVFKNIDGYFKIYSKHTPINLVRDLPQGFSALEPLVDLPIGINITRFQT<br>LLALHRSYLTPGDSSSGWTAGAAAYYVGYLQPRTFLLKYNENGTITDAVDCALDPLSET<br>KCTLKSFTVEKGIYQTSNFRVQPTESIVRFPNITNLCPFGEVFNATRFASVYAWNRKRISN<br>CVADYSVLYNSASFSTFKCYGVSPTKLNDLCFTNVYADSFVIRGDEVRQIAPGQTGKIA<br>DYNYKLPDDFTGCVIAWNSNNLDSKVGGNYNYLYRLFRKSNLKPFERDISTEIYQAGST<br>PCNGVEGFNCYFPLQSYGFQPTNGVGYQPYRVVVLSFELLHAPATVCGPKKSTNLVKN<br>KCVNFNFNGLTGTGVLTESNKKFLPFQQFGRDIADTTDAVRDPQTLEILDITPCSFGGVS<br>VITPGTNTSNQVAVLYQDVNCTEVPVAIHADQLTPTWRVYSTGSNVFQTRAGCLIGAEH<br>VNNSYECDIPIGAGICASYQTQTNSPRRARSVASQSIIAYTMSLGAENSVAYSNNSIAIPT<br>NFTISVTTEILPVSMTKTSVDCTMYICGDSTECSNLLLQYGSFCTQLNRALTGIAVEQDK<br>NTQEVFAQVKQIYKTPPIKDFGGFNFSQILPDPSKPSKRSFIEDLLFNKVTLADAGFIKQY<br>GDCLGDIAARDLICAQKFNGLTVLPPLLTDEMIAQYTSALLAGTITSGWTFGAGAALQIP<br>FAMQMAYRFNGIGVTQNVLYENQKLIANQFNSAIGKIQDSLSSTASALGKLQDVVNQN<br>AQALNTLVKQLSSNFGAISSVLNDILSRLDPPEAEVQIDRLITGRLQSLQTYVTQQLIRAA<br>EIRASANLAATKMSECVLGQSKRVDFCGKGYHLMSFPQSAPHGVVFLHVTYVPAQEKN<br>FTTAPAICHDGKAHFPREGVFVSNGTHWFVTQRNFYEPQIITTDNTFVSGNCDVVIGIVN<br>NTVYDPLQPELDSFKEELDKYFKNHTSPDVDLGDISGINASVVNIQKEIDRLNEVAKNLN<br>ESLIDLQELGKYEQYIKWPWYIWLGFIAGLIAIVMVTIMLCCMTSCCSCLKGCCSCGSCC<br>KFDEDDSEPVLKGVKLHYT

The deleted sequence encompassing the furin cleavage site and double proline substitution in S2 are indicated in red.

Appendix Table S3. Sequences of primers used to genotype B6.K18-hACE2^{IP-THV} transgenic mice.

**Primers**	
hACE2 Fw	TCCTAACCAGCCCCCTGTT
hACE2 Rv	TGACAATGCCAACCA CTATCACT
PKD1 Fw	GGCTGCTGAGCGTCTGGTA
PKD1 Rv	CCAGGTCCTGCGTGTCTGA
GAPDH-ACE2 Fw	GCCCAGAACATCATCCCTGC
GAPDH-ACE2 Rv	CCGTTCAGCTCTGGGATGACC

Appendix Table S4. Sequences of primers used to quantitate SARS-CoV-2 RNA content by qRT-PCR.

Primer/Probe	DNA Sequence
"E-Sarbeco" Fw	5'-ACAGGTACGTTAATAGTTAATAGCGT-3'
"E-Sarbeco" Rv	5'-ATATTGCAGCAGTACGCACACA-3'
"E-Sarbeco"	5'-FAM-ACACTAGCCATCCTTACTGCGCTTCG-BHQ-1-3'
"E-sgmRNA" Fw	5'-CGATCTCTTGTAGATCTGTTCTC-3'
