# Supplementary material for: Brain cross‐protection against SARS‐CoV‐2 variants by a lentiviral vaccine in new transgenic mice
Source: EMBO Mol Med. 2021 Oct 25;13(12):e14459. doi: 10.15252/emmm.202114459 (PMC8646827; doi:10.15252/emmm.202114459)
Supplement: Supplementary file 2 — Expanded View Figures PDF [file EMMM-13-e14459-s001.pdf]

Expanded View Figures

Figure EV1. Inflammation status of the lungs.

A Cytometric analysis of innate cell population and qRT-PCR analysis of cytokines and chemokines in the lungs of LV::S- or sham-vaccinated and SARS-CoV-2-challenged B6.K18-hACE2^{IP-THV} transgenic mice. Cytometric gating strategy to quantify various lung innate immune cells at 3 dpi. Cells were first gated on hematopoietic CD45$^+$ cells and then by sequential gates, through three distinct paths.

B Percentages of selected innate immune subsets versus total lung CD45$^+$ cells were determined in individual mice (n = 6/group).

C Heatmap representing $\log_2$-fold change in cytokine and chemokine mRNA expression in the lungs of LV::S- or sham-vaccinated mice at 3 dpi (n = 6/group). Data were normalized versus untreated controls. Statistical significance was evaluated by Mann–Whitney test (*$P < 0.05$, **$P < 0.01$, ns = not significant).

© 2021 The Authors

© 2021 The Authors

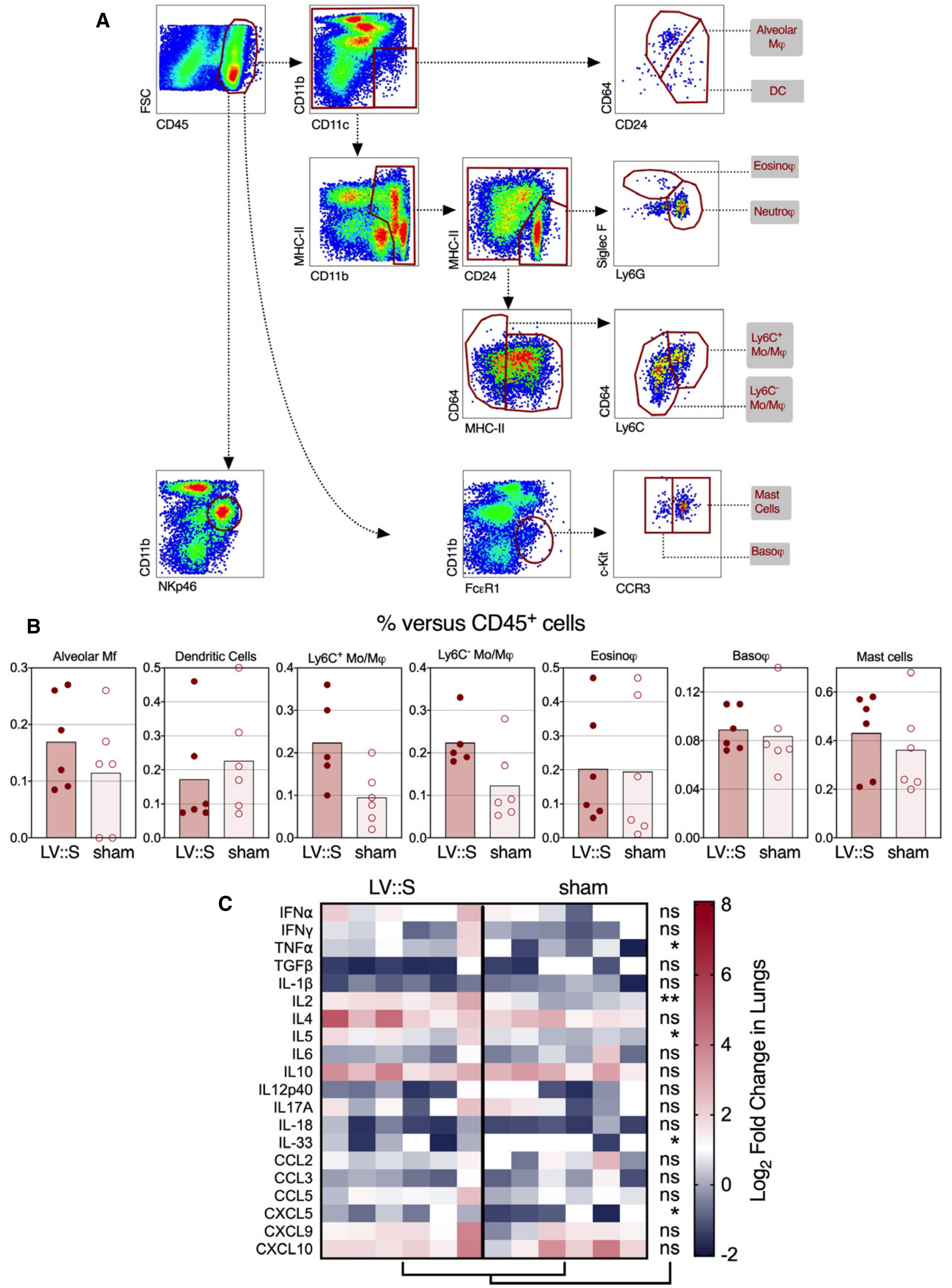

Figure EV1.

© 2021 The Authors EMBO Molecular Medicine 13: e14459 | 2021 23 of 21

Figure EV2. Lung and brain histology in B6.K18-hACE2^{IP-THV} mice, vaccinated with LV::S and challenged with SARS-CoV-2 Gamma.

A, B H&E (rows 1 and 3) and N_{CoV-2}-specific IHC (rows 2 and 4) staining of 3 dpi whole-lung sections from B6.K18-hACE2^{IP-THV} mice, LV::S-, or sham-vaccinated and challenged, following the time line in Fig 7A. H&E and IHC were performed on contiguous sections. Scale bar: 500 μm. The boxed area in the IHC images harbors N_{CoV-2}-specific labeling, as exemplified at higher magnification in (B) Scale bar: 100 μm. They correspond to inflammatory infiltrates seen in the corresponding H&E-stained sections.

C Representative images of N_{CoV-2}-specific IHC staining of whole-brain section in sham or LV::S-vaccinated mice. Boxes highlight clusters of N^{CoV-2} positive cells. Scale bar: 500 μm.

D An example of brain IHC signal at higher magnification and frequency of $N_{CoV2}{}^{+}$ cells in brains from vaccinated and sham animals. Scale bar: 100 μm. Numbers of $N_{Cov2}{}^{+}$ cells per mm^2 of brain were determined in individual mice (n = 5/group). Statistical significance was evaluated by Mann–Whitney test (**$P < 0.01$).

© 2021 The Authors

© 2021 The Authors

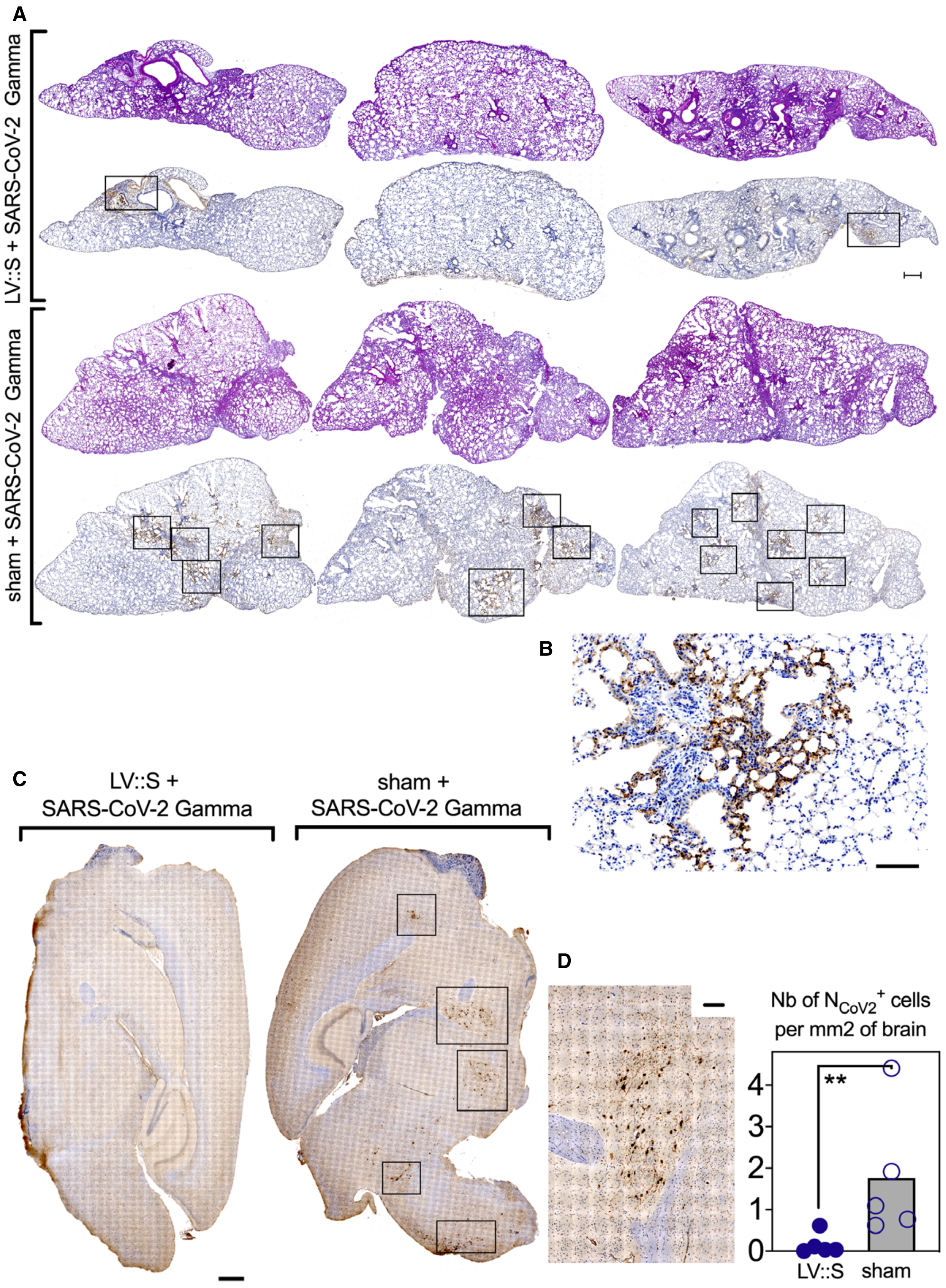

Figure EV2.

© 2021 The Authors EMBO Molecular Medicine 13: e14459 | 2021 25 of 21
