# Supplementary material for: Brain cross‐protection against SARS‐CoV‐2 variants by a lentiviral vaccine in new transgenic mice
Source: EMBO Mol Med. 2021 Oct 25;13(12):e14459. doi: 10.15252/emmm.202114459 (PMC8646827; doi:10.15252/emmm.202114459)
Supplement: Supplementary file 3 — Source Data for Figure 7 [file EMMM-13-e14459-s004.zip › Fig 7-Source Data.pdf]

Please find source data of the histological analyses included in the Figure 7:

Figure 7A Top: This Figure has been already included in the first submission. As required by one reviewer, we added Fig 7A Bottom, which is a representative experimental control.
Figure 7A Right: Compared to the first submission, we also extended the analysis to more mice/group as asked by one of the reviewers.

Figures 7B, C, D are the same cytometric analyses included in the first submitted version.

Figure 7E: The four histological figures have been included in the first submitted version. While preparing the revised version, as required in EMBO MM Instruction to Authors, we removed the values describing the size of the scale bars from the images and added these values to the legend to the Figure. Is this the potential aberration that you detected?

Figure 7A

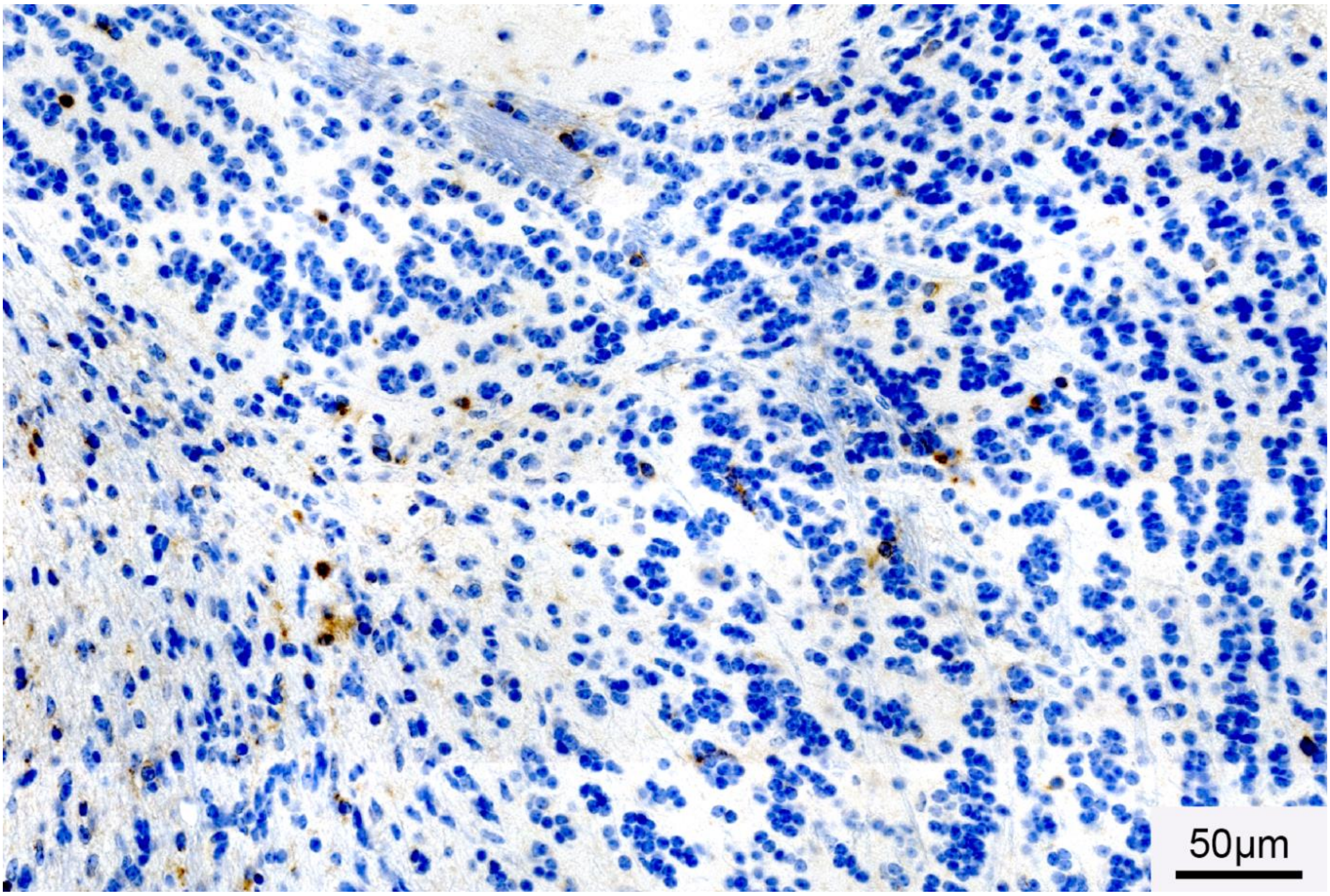

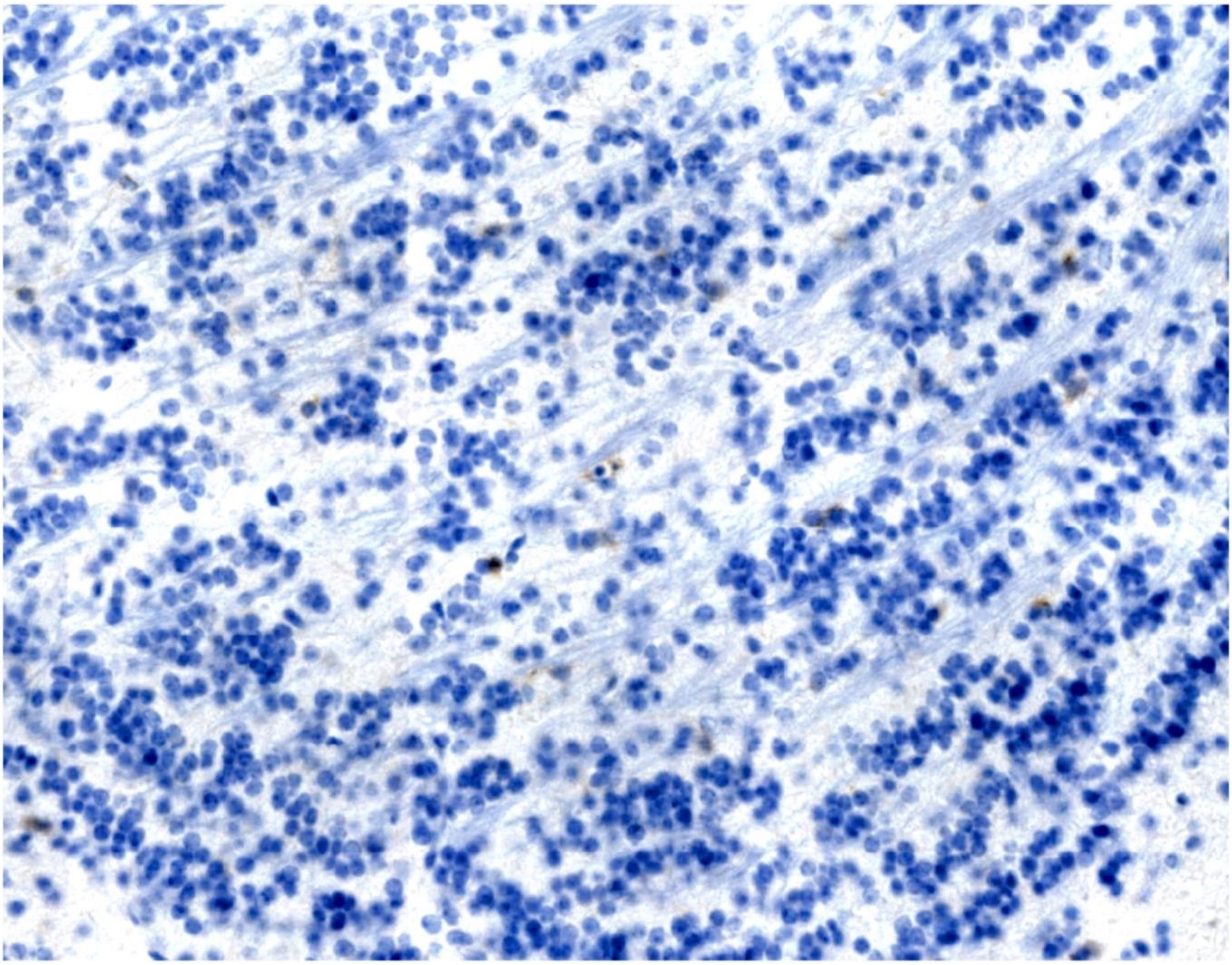

Figure 7E

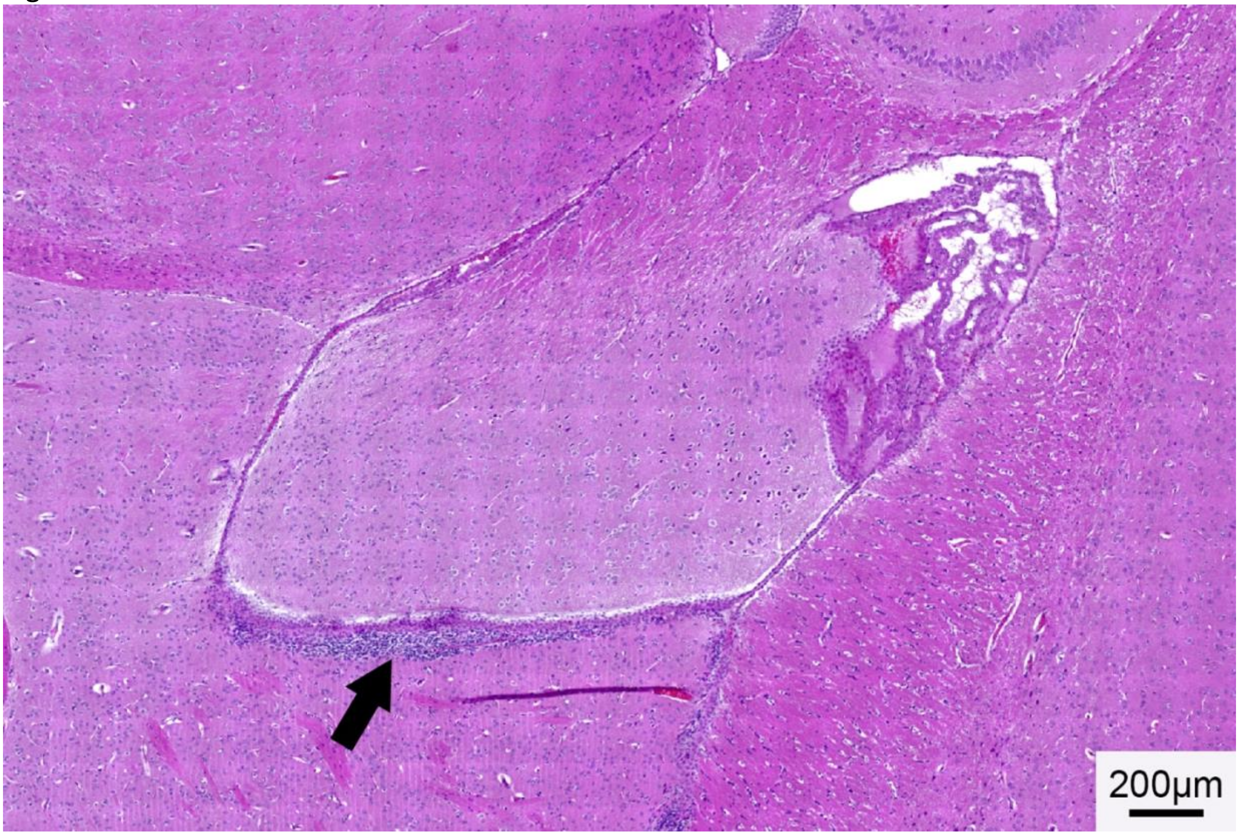

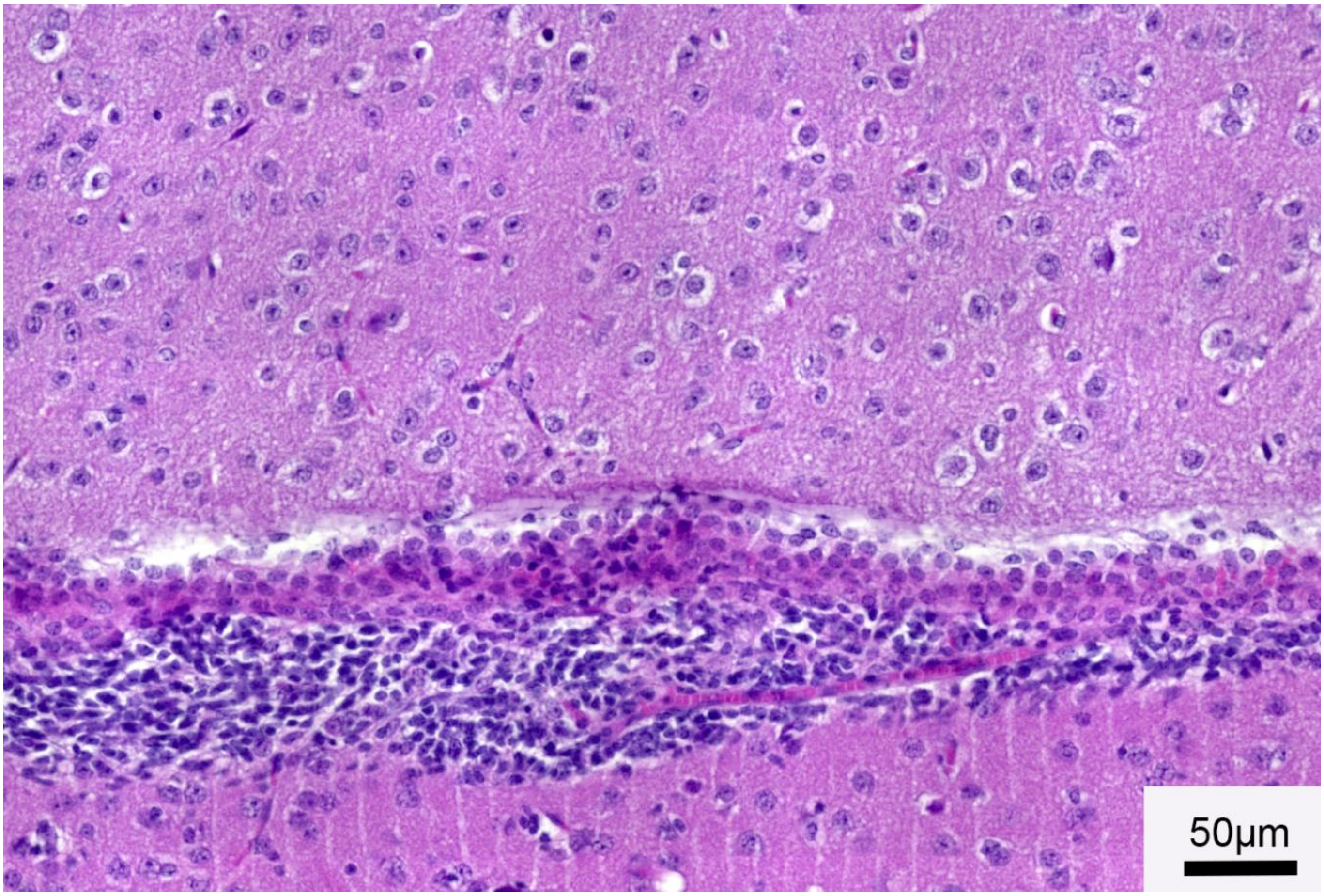
50μm

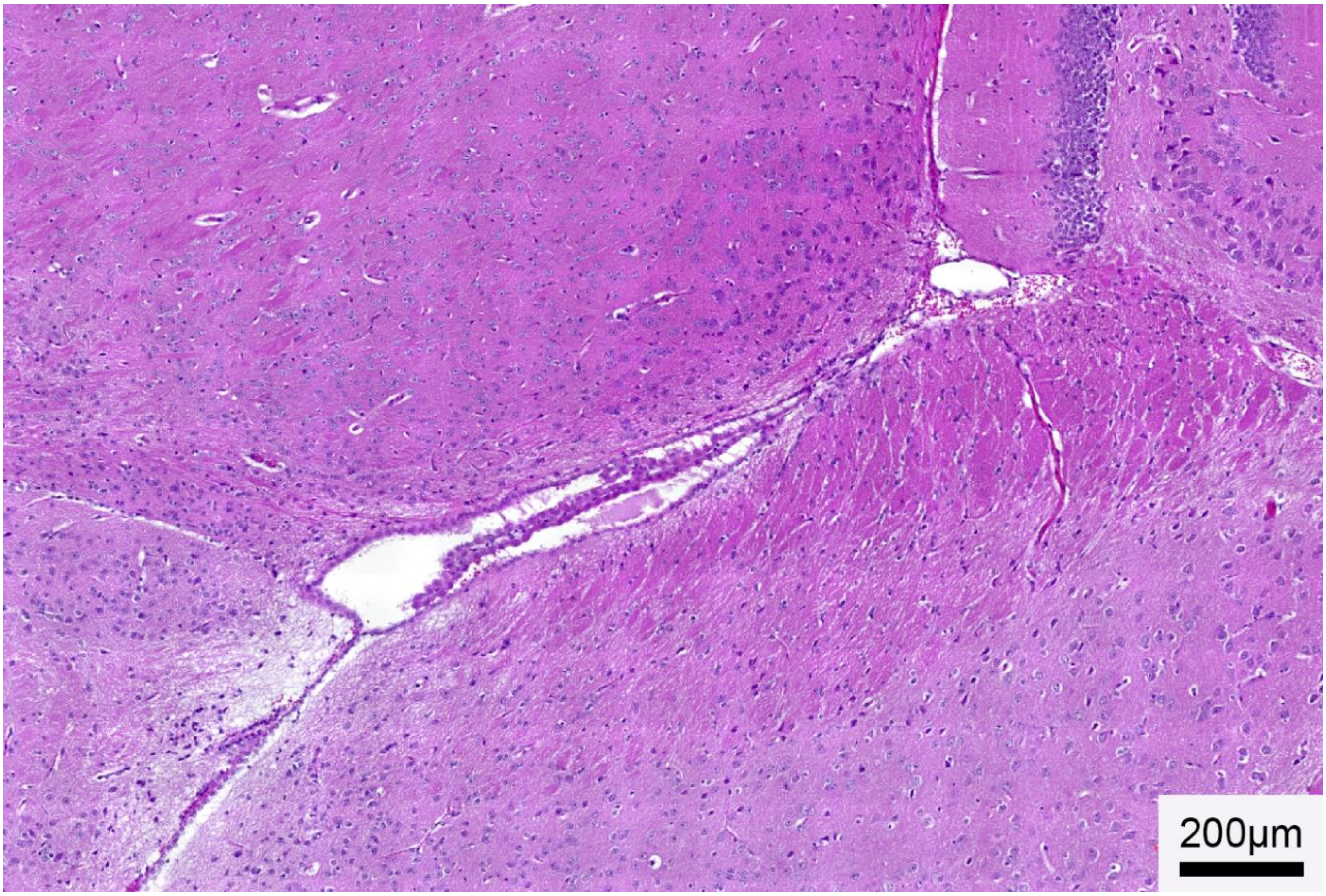
200μm

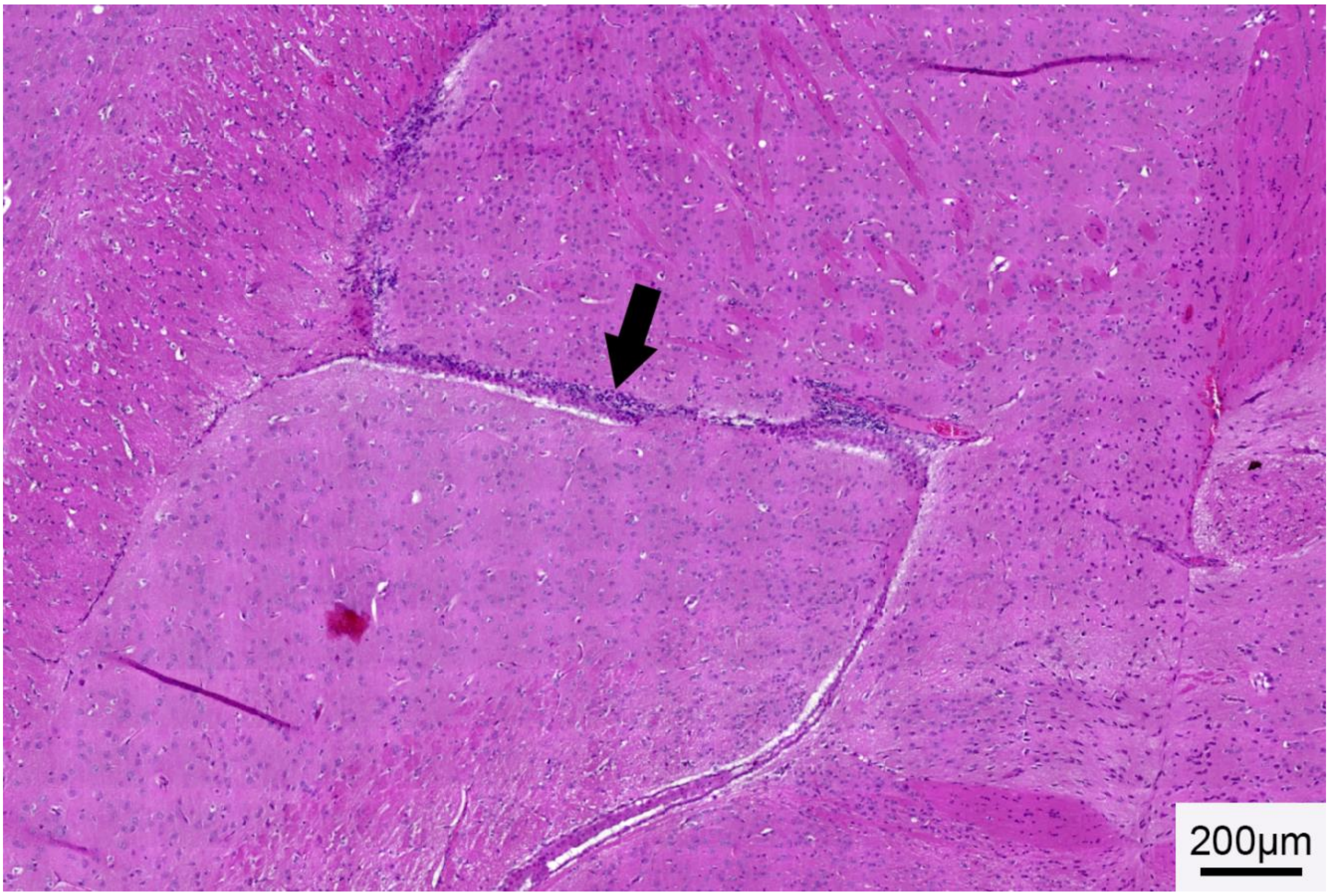
200µm
